# Compositional and seasonal differences of gas and particle phase polycyclic aromatic hydrocarbons (PAHs) over the southern Baltic Sea coast

**DOI:** 10.1038/s41598-022-25666-5

**Published:** 2022-12-05

**Authors:** Patrycja Siudek

**Affiliations:** Institute of Meteorology and Water Management, Waszyngtona 42, 80-342 Gdynia, Poland

**Keywords:** Climate sciences, Environmental sciences, Health care

## Abstract

In this study, 16 USEPA-prioritized PAHs in gas- and particle-phase (PAH_*g*+*p*_), associated chemical and meteorological parameters, and backward trajectory simulations were explored in a coastal city in Poland, between April 2019 and May 2020. This study reports several important aspects of PAH_*g+p*_, i.e. variation, composition, distribution profiles, impact of weather conditions, and correlation analysis between target PAH compounds and influencing inorganic gaseous pollutants. Specifically, higher and more variable concentrations of total PAH_*g*+*p*_ (mean ± SD, ng m^−3^) were observed during winter (36.38 ± 24.19) compared to autumn (22.3 ± 17.44), summer (21.52 ± 13.30) and spring (19.90 ± 13.13). A distribution profile of parent PAH_*g*+*p*_ was as follows: 3-ring > 4-ring > 2-ring > 5-ring > 6-ring, although their relative contribution to the total PAHs showed statistically significant differences between seasons (*p* < 0.05). Precipitation-driven loss of ΣPAH_*g*+*p*_ was lower in the warm period than in the cold one, reflecting higher PAH concentrations in winter. A seasonal model-based analysis of incremental lifetime cancer risk showed a higher potential cancer risk for children than those for adult females and males. The adverse health impacts associated with PAH exposure via inhalation route indicate the need for implementation of pollution-control policies in this region.

## Introduction

Parent polycyclic aromatic hydrocarbons (PAHs) are key semivolatile organic components of the atmospheric system that have a well-documented influence on air quality, global climate change and health implications^1–3^. Anthropogenic sources of PAHs are mainly related to emissions from incomplete combustion of fossil fuels in commercial (i.e. power plants, industrial activities) and residential sectors (i.e. use of boilers, stoves, heating units), biomass burning (biofuels), vehicle emissions, oil spills, and ship emissions^4^. The major non-urban origins of PAHs are wildfires and soil/water system emissions. Sources of PAHs can be structurally identified and apportioned based on different methods, i.e. positive matrix factorization^5,6^; diagnostic ratios^7–11^; principal component analysis^12,13^; and hybrid receptor models^14^. Many other studies recognized that other environmental parameters should be examined to address the seasonal variability, levels and processes of semivolatile aromatic hydrocarbons^15^. Recently, scientific evidence from various observations suggested that interactions with meteorology (i.e. ambient temperature, relative humidity, wind regime, atmospheric stratification), and inorganic gaseous pollutants largely influence PAH fate, mass distribution, and biogeochemical cycling^16^. In addition, PAHs can be involved in a variety of complex homo- and heterogeneous atmospheric chemical reactions. Measurements during the past decades outlined the key role of radicals (OH^·^, HO_2_^·^, NO_3_^·^) in the atmospheric chemistry of PAHs on local, regional and global scales. These reactive species could affect PAH physico-chemical properties, gas-to-particle partitioning, distribution, changes in temporal scales, and a variety of aging processes (e.g. photochemical and oxidative)^16,17^.

Precipitation is an important driver of PAH atmospheric chemistry. Previous studies^15,17^ highlighted that precipitation regime (type, rate, intensity, frequency) significantly affects PAH profiles and levels in the ambient air. Wang et al*.*^18^ found that daily light rainfalls in the range of 1–20 mm play a crucial role in atmospheric particles’ wet deposition rates. As reported in Omokpariola et al*.*^19^, atmospheric rainwater directly affected PAH spatiotemporal variability and budget in heavily polluted areas in Nigeria. These findings indicate that precipitation can serve as a good marker of PAH sources, distribution, transformation and deposition pathways.

Many epidemiological studies demonstrated clear links between serious human health problems (i.e. respiratory illnesses, lung cancer) and high levels of carcinogenic PAH congeners in inhaled air. For example, increased health risks posed by toxic airborne compounds were reported close to highly polluted hotspots in China^2,5^, India^4^ and Iran^20^. However, only limited number of these studies considered both the gas and particle phases while estimating incremental lifetime cancer (ILCR) risk from exposure to toxic PAHs, separately for adults and children.

Long-term monitoring of the gas- and particle-phase PAH compounds is also crucial for studying their various sources and transformation mechanisms in the coastal regions under global climate change. It should be noted that complex PAH measurements including source apportionment and seasonal distribution, are still very limited in central European coastal and offshore areas (i.e. Baltic Sea, the Danish Straits). Over southern Europe (i.e. Mediterranean Sea, Black Sea, North Sea, Arabian Sea)^21,22^, only a few studies examined the contribution of shipping-related PAH inputs. So far, PAH-oriented studies in Poland have been limited to PM_10_ and PM_2.5_ fractions and urban regions i.e. Silesia^23,24^, Poznań^25^, and Tricity Agglomeration^26,27^. However, none of these studies directly addressed the comprehensive investigation of PAH concentrations measured simultaneously in gas and particle phases, their composition and distribution, potential human exposure, and adverse health effects.

In this study, results from the intensive field campaigns focused on 16 parent PAHs in the gas and particle phases (PAHs_*g*+*p*_) performed at a coastal city in Poland are presented.

The primary objectives were as follows:

**#**1 to characterize the molecular distribution of PAHs_*g*+*p*_,

#2 to analyze PAHs_*g*+*p*_ seasonal profiles,

#3 to identify correlations between individual PAHs_*g*+*p*_ and inorganic gas pollutants (NO_×_, O_3_, SO_2_, NO_2_, CO),

#4 to examine PAHs_*g*+*p*_ trends related to basic meteorological parameters (T, Rh, V_s_, V_d_),

#5 to characterize the effects of precipitation on the total PAHs_*g*+*p*_ budget,

#6 to evaluate human exposure to carcinogenic PAHs and health impacts based on BaP equivalent concentration (BaP_**eq**_) and incremental lifetime cancer risk (ILCR).

This work highlights the role of factors affecting the seasonal variability of individual PAH congeners and their relation to gas precursors. Results from this study fill a data gap and this is crucially important for a better understanding of changes in PAH composition and distribution in the Polish coastal urban region of the southern Baltic Sea.

## Methods

### Site characteristics

Measurements of gaseous and particulate PAHs were made at the monitoring station in Gdynia (long. 54° 52′ N, lat. 18° 56′ E) between April 2019 and May 2020 (n = 112). Gdynia is a large coastal city in Pomorskie Province (N Poland), with a population of 250 000. This city is characterized by transitional and warm summer continental climate with four main seasons: spring (March–April–May, MAM), summer (June–July–August, JJA), autumn (September–October–November, SON) and winter (December–January–February, DJF). It represents a typical urban-mixed area, surrounded by a harbor infrastructure (i.e. port and docks, ship repair yard), buildings (i.e. commercial/residential, domestic heating units, coal-fired heating boilers), and relatively high traffic density. Additionally, major industrial point sources such as petrochemical refineries, steel manufacturing, coal-fired power plants, and municipal solid waste recycling units are mainly located on the west and southeast of the sampling site (Fig. S1). Furthermore, it should be noted that coal combustion is a major source of atmospheric pollution in this region^28^. Thus, Gdynia's air quality is particularly poor in winter because of the influence of different local/regional anthropogenic emissions and air masses transported from other severely polluted areas in Poland and continental Europe (i.e. France, Germany, Netherlands, Czech Republic). Previous backward trajectory analysis revealed that elevated winter PAH concentrations in PM_2.5_ were frequently detected during transport from southerly and southwesterly flows^29^.

### PAH measurements

The sampling system was placed on the rooftop of the two-stored building (20 m above the ground). The offline filter-based approach was used to simultaneously collect PAHs in the gas and particle phases using Low Volume manual sampler (Comde Derenda, Germany). The instrument was equipped with an integrated PUF-PM impactor to pump air and collect separately gas and PM_10_ phase samples (aerodynamic diameter of < 10 µm) at a target flow rate of 2.30 m^3^ per hour. Briefly, particulate PAHs were collected onto Quartz filters (47 mm in diameter, QM-A, Whatman), while gas phase was collected onto polyurethane foam PUF plugs (6 cm OD × 5.1 cm length, Restek). Measurements were done over 24 h period, and 16 samples (8 gas + 8 particle phase) were collected each month between April 2019 and May 2020 (14 months giving 112 sampling days).

### Sample pre-treatment and extraction

Before the field sampling, the QM-A filters were pre-baked in a muffle furnace (550 °C, 8 h) to eliminate residual organic impurities. The QM-A filters were conditioned in a borosilicate glass desiccator at 24 °C and 40% RH for at least 24 h to avoid exposure to ambient air and then weighted at a microbalance with 10 µg precision. PUF plugs were at first double pre-extracted in acetone and *n*-hexane (1:1, v/v, Merck), following the extraction in dichloromethane:*n*-hexane (1:1, v/v, Merck) as mobile phase. This step was repeated 2 times for each sample. The extraction was performed in ASE350 Accelerated Solvent Extractor (Dionex, Thermo Fisher Scientific) using the following program: heating to 100 °C for 5 min (the pressure was maintained at 1500 psi and remained static for 15 min), then the system was cooled down to ambient temperature. The pre-cleaned PUFs were then rigorously dried in a vacuum system, wrapped into two layers of aluminum foil, placed into zip-lock polyethylene bags, and stored in low temperature-controlled conditions until sampling.

Immediately after each one-day sampling, PUFs and QM-A filter samples were transported to the laboratory. After field exposure, filters were conditioned for 24 h in a desiccator, weighted, and then enveloped in aluminum foil, and stored frozen at − 20 °C, while PUFs were directly placed into zip-lock bags and kept frozen until chromatographic analysis. This field measurement strategy resulted in 250 samples for chemical analysis, including PUF and filter blanks from each sampling period. In the current study, the total PAHs are the sum of the gas and particle phases (PAHs_*g*+*p*_).

Before the main analysis, all samples (PUF plugs and QM-A filters) were extracted using the same procedure. In brief, samples were placed in ASE extraction cells containing each a cellulose filter at the bottom. The extraction was performed at 100 °C and 1500 psi for two 20 min cycles using an ASE350 instrument (Dionex, Thermo Fisher Scientific) and a solvent mix of dichloromethane (DCM) and *n*-hexane (1:1 in volume, Merck). The extracts were cleaned up by quantitatively transferring them to the glass chromatography column filled with glass wool, 1 cm of anhydrous Na_2_SO_4_, 2 g of 2% activated silica gel, 1 cm of anhydrous Na_2_SO_4_ on top, and target analytes were eluted with 20 mL of DCM and *n*-hexane mixture (1:1 in volume). After that, purified eluates were concentrated on the rotary evaporator to about 2 mL and evaporated to 0.5 mL under a gentle stream of nitrogen in a 35 °C water bath. Final extracts were transferred into scaled glass ampoules and stored in a refrigerator at 4 °C for chromatographic analysis. All the glassware was rigorously prepared, i.e. washed with neutral cleaning foam and tap water, rinsed with purified deionized water (Millipore system at 18.2 MΩ), and dried at 400 °C for 2 h to remove any organic residuals.

### PAH analysis

The method for PAH analysis has been described in our previous studies^34,35^. Concentrations of PAH congeners in gaseous and particulate fractions were determined using liquid chromatography (HPLC Shimadzu Prominence) with fluorescence (15 PAHs) and diode-array detection (254 nm, used for Acy quantification due to its weak fluorescence). The instrument consists of an RF-20A/RF-20Axs fluorescence detector, CTO-20A/20AC column oven, LC-20AD prominence HPLC pump, online degassing unit DGU-20A, and system controller CBM-20A, autosampler Sil-20A, UV–Vis detector SPD-20A, and a photodiode array detector SPD-M20A.

About 25 μL of the final extracted sample was injected automatically into HPLC-FLD/UV. The separation was performed on a Kinetex LC column (150 mm × 4.6 mm i.d., particle size of 3.5 μm, Phenomenex). The individual PAHs were separated using gradient elution at a flow rate of 0.8 ml min^-1^, with acetonitrile:deionized water mixture (1:1, *v*/*v*) as a mobile phase. The extinction and emission wavelength were as follows: Nap–Phe: excitation Ex, λ = 270 nm, emission Em, λ = 350 nm; Flu–Pyr: Ex, λ = 250 nm, Em, λ = 420 nm; BaA–Chry: λ = 270 nm, Em, λ = 390 nm; BbF–DahA: λ = 290 nm, Em, λ = 430 nm, BghiP–IcdP: λ = 360 nm, Em, λ = 460 nm.

### Quality assurance/quality control for PAHs analysis

Calibration was performed using a six-point calibration curves covering the concentration range 0.05; 0.02; 0.03; 0.05; 0.10; 0.15 ug/ml (16 EPA Priority PAH Mix, 10 ug/ml in acetonitrile, Chiron, S-4065-10-5AN). The limit of detection for PAHs expressed as three times the standard deviation of laboratory blanks were in the range of 0.001 ng m^−3^ (BaA) and 0.016 ng m^−3^ (DahA). Field and laboratory blanks were measured following the same procedure as in the case of ambient samples to monitor the levels of target PAHs (6 PUFs and 6 filters) and their possible contamination throughout the whole sampling procedure. The results for field blank filter samples were much lower than those of environmental samples and represented less than 7% of the quantified values. PUF samples were corrected for blank by subtracting the pre-cleaned PUF values from environmental samples.

A certified reference material *ERM-CZ120 Fine dust* was used for verification of the method. Briefly, blank filters were loaded with 0.5 mg of CRM for target PAHs quantification (n = 10). The recovery of aromatic hydrocarbons from *ERM-CZ120* was between 82 and 110%. Additionally, clean blank PUF (n = 6) and quartz filters (n = 6) were spiked with 25 µl of the surrogate standard at 50 µg ml^-1^, containing 1-fluoronaphthalene (Chiron, 1313.10-100-AN), and 2-fluorophenantrene (Chiron, 1328.14-10-AN) to quantify recovery yields. These samples were extracted and analyzed in the same manner as environmental samples. The average recovery levels of surrogate standards were relatively good (71–89%).

### Auxiliary data

In this study, direct measurements of basic meteorological parameters, including the ambient temperature and relative humidity, wind speed and direction, precipitation amount, and gas species data (CO, NO_×_, NO_2_, SO_2_, O_3_) were registered using local air quality and weather station data acquisition systems (i.e. IMGW, ARMAAG, WIOŚ). During the cold season (Jan, Feb, Oct–Dec) the prevailing wind was between south-west to westerly (mean speed of 4.62 m s^−1^), while westerly winds were identified as dominant during the warm season (mean speed of 3.91 m s^−1^).

### Health impact analysis

The benzo(a)pyrene toxicity equivalent of bulk carcinogenic PAHs (i.e. BaA, BbF, BkF, BaP, DahA, BghiP, IcdP) was calculated using the following Equation:

1 $$BaP_{eq} \left( {ng \, m^{ - 3} } \right) = \mathop \sum \limits_{i = 1}^{n} PAH_{i} \times TEF_{i}$$

where *PAH*_*i*_ is the concentration of individual carcinogenic PAH (ng m^−3^), *TEF*_*i*_ is the toxic equivalency factor for each PAH compound according to reference data by Nisbet and Lagoy^30^ provided in Table S1.

The lifetime average daily dose (*LADD*) and the incremental lifetime cancer risk (*ILCR*) of toxic PAH compounds are common parameters of health risk via the inhalation route proposed by many epidemiologically based models^4,7,31–35^. In the present study, to calculate the public health risk due to long-term and continuous exposure to carcinogenic PAHs in this study, the following Eqs. (2 and 3) are used:

2 $$LADD \; \left( {{\text{mg}}\;{\text{ kg}}^{ - 1} \;{\text{day}}^{ - 1} } \right) \, = \, Cs \, \times \, IR \, \times \, CF \, \times \, EF \, \times \, ED{/}\left( {BW \, \times \, AT} \right)$$

3 $$ILCR \; \left( {unitless} \right) \, = \, LADD \, \times \, CSF$$

where *Cs* is the sum of PAH concentrations calculated by multiplying the concentration of individual PAH and their corresponding toxic equivalency factors (TEF) in gas and particle phases (ng m^-3^), *IR* is the air inhalation rate (m^3^ day^-1^), i.e. 15.2 for adults and 10.0 for children, *CF* is the unit conversion factor (1 × 10^−6^ mg kg^-1^), *EF* is the exposure frequency (day year^-1^), *ED* is the lifetime exposure duration of 6 years for children and 70 years for an adult, *BW* is the body weight (kg), i.e. 65 kg for adult females, 85 kg for adult males, and 10 kg for children, *AT* is the average lifetime for carcinogens (days), (70 years × 365 days year^-1^), *CSF* is the inhalation cancer slope factor (3.85 mg kg^-1^ day^-1^).

Moreover, the exposure of atmospheric PAHs via the inhalation route was examined for different population groups, including children, adult female/male, and is shown separately for the cold (SON + DJF) and the warm (MAM + JJA) seasons. It should be noted that only PAHs that are well known for their carcinogenic, mutagenic and teratogenic activities (i.e. compounds with TEF values higher than 0.001) were considered in this study. This is well in line with other previous epidemiological methodologies to screen the potential human risks and assemble results into age packages^31,32^.

## Results and discussion

### Concentration levels, compositions and intra-annual variation of gas and particle PAHs

**Figure 1:**
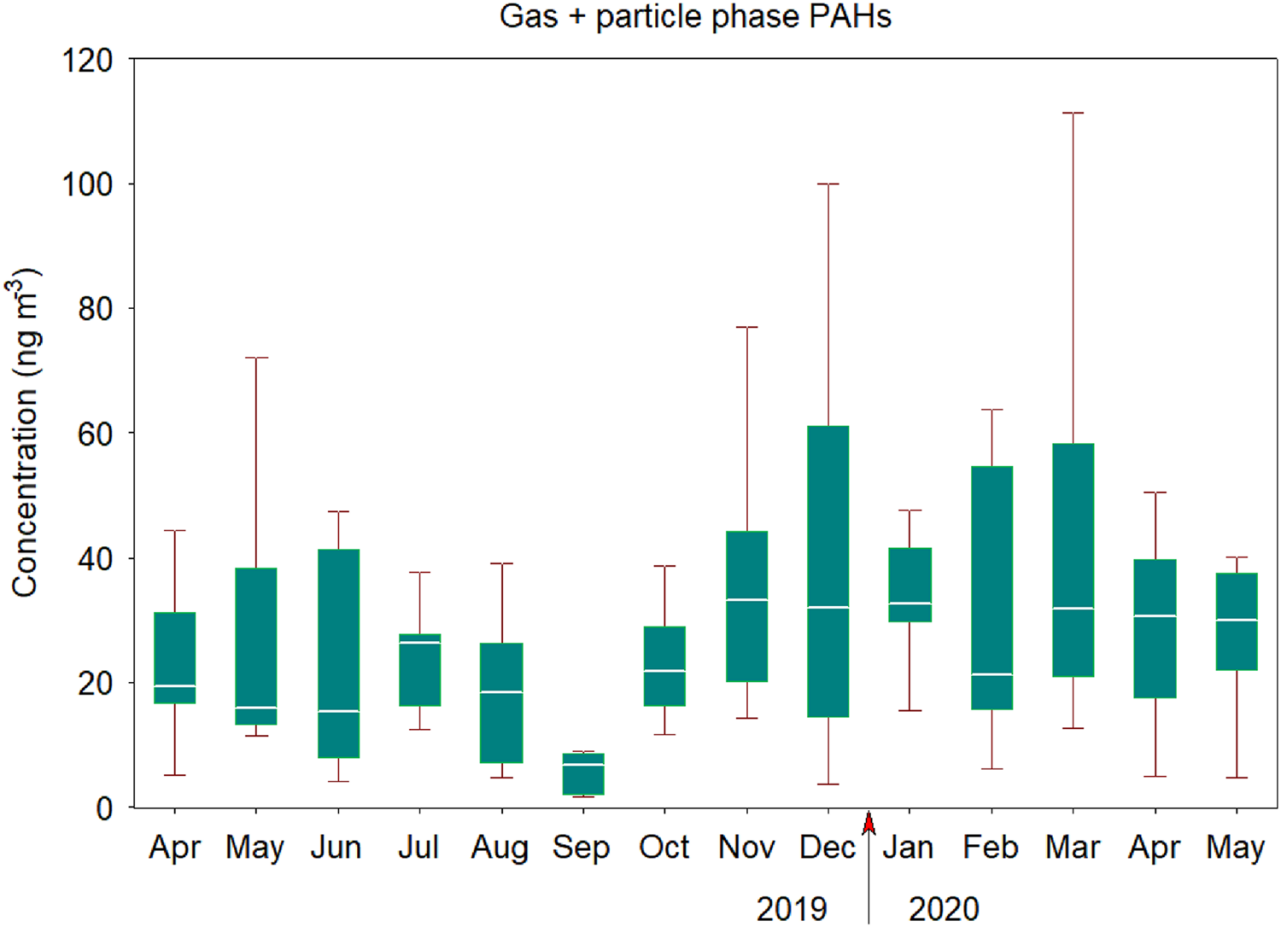
Boxplot of Σ_16_PAHs concentrations in the gas and particle phases during the 2019–2020 sampling period in Gdynia, Poland. The boxes represent the 25th and 75th quantiles, the black line inside represents the median value, and the bottom/top whiskers are the minimum and maximum values, respectively.

The monthly concentration of Σ_16_PAHs_*g*+*p*_ in collected samples showed considerable variability (Fig. 1). The annual mean value of Σ_16_PAHs_*g*+*p*_ was 27.99 ± 20.01 ng m^−3^. The lowest daily concentration of the total PAHs_*g*+*p*_ amounting to 1.78 ng m^−3^ was found in September 2019, while the highest concentration of 111.30 ng m^−3^ was observed in March 2020 (Fig. S2). It is interesting to note that the peak concentration of total PAHs_*g*+*p*_ in March was slightly higher than in December 2019 (100.03 ng m^−3^), reflecting a relatively high contribution of 2-, 3- and 4-ring compounds to daily ΣPAHs_*g*+*p*_ under more stagnant conditions.

Generally, concentrations of ΣPAHs_*g*+*p*_ measured in this study were comparable with results from Prague in the Czech Republic^36^, higher than levels observed in remote mountain regions of Europe, i.e. Spain, Austria, Norway^37^ and China^15^, but lower than those registered in urban Bursa, Turkey^8^, Guangzhou, and Beijing in China^38,39^, and Agra in India^40^. Much higher levels of PAHs, as compared with results from this study, were observed in the Middle East, especially in Giza, Egypt^41^. On the contrary, a recent ship-borne AQABA project^22^ reported distinct regional patterns in PAHs trends, showing substantially lower concentrations of parent PAHs in the gas and particle phases over the Mediterranean Sea, the Red Sea, the Arabian Sea, the Gulf of Oman, and the Arabian Gulf than those determined over coastal Chinese regions^42^ and the Baltic Sea^43^. Specifically, the mean concentration of Σ_26_PAHs in the coastal marine atmosphere of southern Europe was 2.99 ± 3.35 ng m^−3^, with the highest values registered in the Mediterranean Sea (mean 4.40 ng m^−3^) and the lowest in the Arabian Sea (mean 0.59 ng m^−3^). Moreover, this study^22^ also found that Phe was the most abundant parent PAH, followed by Flu, Ace, Flt, and Pyr, with mean contributions of 24%, 10%, 6%, and 5%, respectively.

**Figure 2:**
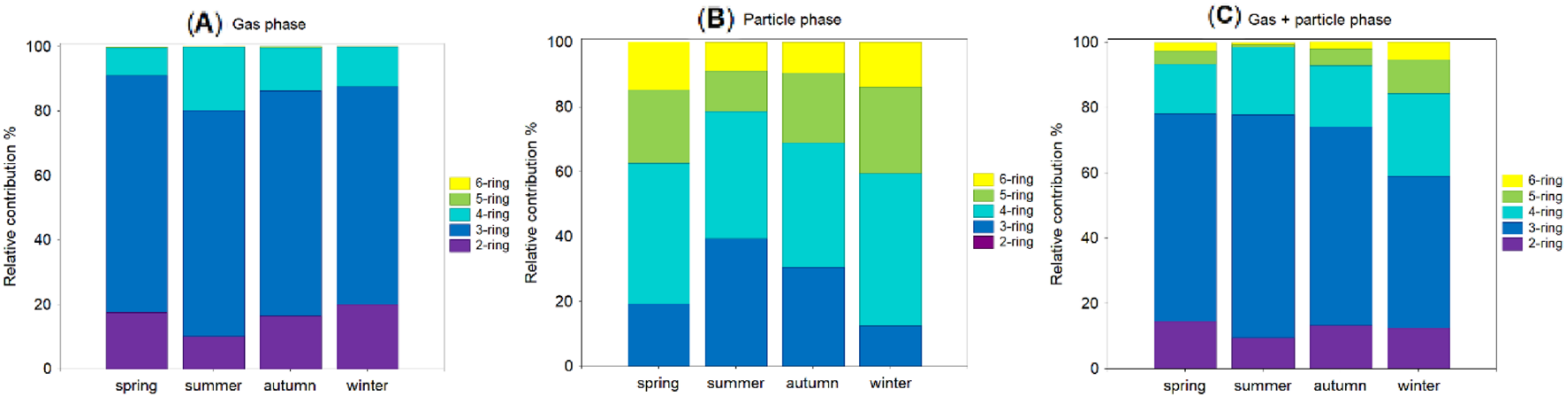
The relative contribution (%) of the five PAH groups (2-, 3-, 4-, 5-, and 6-ring) to the total PAHs in (**A**) the gas phase, (**B**) the particle phase, and (**C**) the gas + particle phases.

The seasonal differences in PAH profiles expressed as various ring-based group compounds in the gas-, particle-phase, and the gas + particle phases are presented in Fig. 2. Measurements revealed the presence of 2-ring and 3-ring PAHs as major isomers in the gas phase, while 5-ring and 6-ring compounds mainly contributed to the particle phase. The differences between 2- and 6-rings PAH distributions were statistically significant (*p* < 0.05). Specifically, the mean mass proportion of 4-ring compounds (Flt to Chry) to the total PAHs ranged from 47% (winter) to 38% (autumn) in the particle phase (Fig. 2B). On the contrary, the contribution of 4-ring PAHs to total PAHs in the gas phase accounted for 9%, 12%, 13%, and 20% in spring, winter, autumn and summer, respectively (Fig. 2A). The sequence of increasing 2-ring PAH contribution to the total PAHs in the gas phase was as follows: summer < autumn < spring < winter (Fig. 2A).

**Figure 3:**
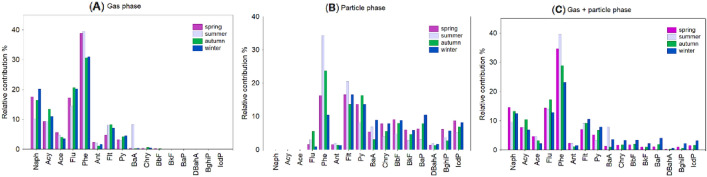
Mean relative contribution (%) of (**A**) the gas PAHs, (**B**) the particle PAHs, and (**C**) the gas + particle phase PAHs during the sampling period.

In the current study, corresponding 3-ring compounds (Acy, Ace, Flu, Phe, Ant) were the predominant contributors to the total PAHs, with a mean percentage of 73% in the gas phase during spring, followed by summer (70%), autumn (70%) and winter (67%). However, 3-ring PAHs in particulate size fraction revealed slightly different trend, i.e. 13% < 19% < 29% < 39% for winter < spring < autumn < summer, respectively (Fig. 2B). A pronounced contribution of Phe to the total PAHs can be found in summer and is displayed in Fig. 3C. It should be noted that previous studies have found a relatively high contribution of 3-ring compounds to the total PAHs in the coastal regions and offshore^22,44–46^. As shown in Fig. 2B, the mass distribution of 5-ring compounds (BbF to DahA) exhibited a clear seasonal pattern in the particle phase, with a considerably higher contribution in winter (27%) than in summer (12%). Considering a 5-ring PAH—benzo(a)pyrene in particulate fraction, it can be seen that it revealed a substantially higher contribution to the total PAHs during winter (10%) compared to summer (1%). The mean contribution of 6-ring PAHs (BghiP, IcdP) in spring and winter was 15% and 14%, respectively, while in summer and autumn 9% and 10%, respectively (Fig. 2B). The observed differences are likely due to a combination of seasonally varying meteorological parameters (i.e. air temperature, relative humidity, precipitation, photo-oxidation, gas/particle partitioning) and emission rates (i.e. heating activities in commercial and residential sectors).

**Figure 4:**
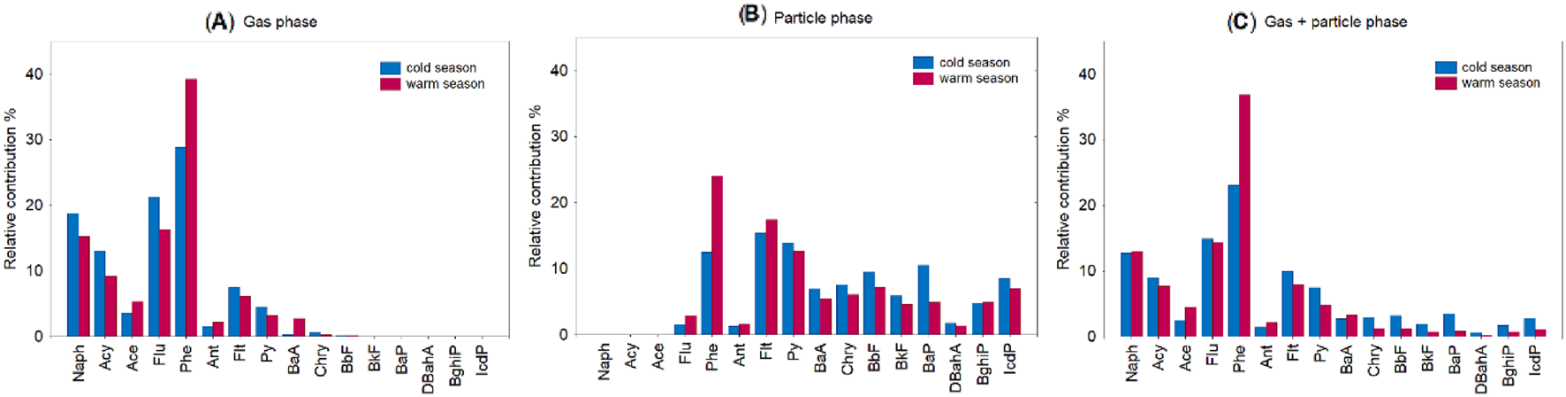
Comparison between two periods (warm and cold) for PAH congener contributions (%) related to (**A**) gas phase, (**B**) particle phase, and (**C**) gas + particle phases.

In this study, carcinogenic PAHs (BaA, Chry, BaP, BbF, BkF, DahA, BghiP, IcdP) were mostly present in the particle phase (Fig. 3). Their contribution to the total PAHs in coarse particulate matter ranged from 1 to 9% in spring, 2–7% in summer, 1–8% in autumn, and 2–10% in winter. The seasonal differences of medium- (BaA, Chry) and high-molecular-weight PAHs (BaP, BbF, BkF, DahA, BghiP, IcdP) would indicate that PM_10_-bound PAHs originated from primary emission sources (i.e. coal combustion, biomass burning, traffic-related emissions, industrial activities) that are variable during the whole sampling period^29^. As shown in Fig. 4, higher contribution of 4–5-ring (i.e. from Pyr to BaP) in PM_10_ fraction was observed during the cold season compared to the warm season, implying that gas-to-particle partitioning could be important for PAH transformations towards the particulate fraction. Esen et al*.*^8^ also reported that high molecular weight congeners revealed a higher contribution to total PAHs during the cold season than in the warm season, which provides a useful comparison for our measurements.

In this study, the diagnostic ratios of selected congeners i.e. BaA, Chry, Flt, Pyr, BaP, BghiP and IcdP were used to roughly distinguish pyrogenic and petrogenic sources. Figure S3 shows season-to-season differences in 5 ratios widely reported in the literature ^7–11^. Following the work by Urbančok et al*.*^7^, the Flt/(Flt + Pyr) and IcdP/(IcdP + BghiP) ratios higher than 0.5 can be good tracers of aged particles originating from combustion processes, including coal, fossil fuels, wood and grass. During the study period, the mean value of Flt/(Flt + Pyr) in the warm season (0.58) was slightly higher than in the cold season (0.55). In contrast, the IcdP/(IcdP + BghiP) ratio ranged from 0.41 to 0.98 (mean = 0.64) in cold season, while lower values were reported in warm season (range: 0.42–0.68, mean = 0.57). These findings suggest that medium- and high-molecular weight congeners originated mainly from local combustion of wood, hard and brown coal as well as biomass burning. A previous study^47^ showed that the IcdP/BghiP ratio > 1.48 is indicative of brown coal combustion in residential and industrial sectors, which is in good agreement with winter observations from this study (Fig. S3). Samples collected in Gdynia showed a slightly higher BaA/(BaA + Chry) ratio during the warm season (0.54) than in the cold study period (0.42), suggesting mixed sources i.e. fossil fuel combustion and traffic emissions. BaA/(BaA + Chry) ratio > 0.5 can be also applied to discriminate local emissions from regional transport, as previously observed by Khalili et al*.*^48^. It was found that the regional transport of air pollutants contributed more to the PAH burden during the cold season than in the warm season (Fig. S3). In this study, the BaP/BghiP ratio was more than 2 times higher during the cold season compared to the warm one. Therefore, vehicular exhaust emission is expected to play an important role in high-molecular-weight PAH pools, depending on season and meteorological conditions, as observed in many previous studies^4,6,7,11,49^.

### Correlation analysis between PAHs, meteorological parameters, and gas species

Table S2 shows Spearman’s rank correlation analysis (*Rs*) performed to assess the statistically significant dependences between concentrations of individual PAH isomers in the gas (only Naph, Acy, Ace) and particle phases, and other parameters at the 95% confidence level (*p* < 0.05). In general, each target PAH compound had a negative correlation with ambient temperature (Table S2). The 2-ring and 3-ring PAHs showed weak to moderate negative correlations with ambient temperature, while correlations for 5-ring PAHs were slightly higher (from − 0.61 to − 0.66, Table S2). The strongest negative correlation coefficient between T and individual isomers was found for Pyr (R*s* =  − 0.71) and Chry (R*s* =  − 0.70), suggesting that their concentrations tend to increase when air temperature decreases. Moreover, ambient temperature shows a negative correlation with CO (R*s* = − 0.64) and SO_2_ (R*s* = − 0.63), indicating contribution from local primary sources such as fossil fuel combustion and biomass burning. Another meteorological parameter that influenced PAH distribution was the precipitation amount (Table S2), with the correlation coefficient ranging between − 0.30 (Ant) and 0.12 (Acy). The impact of precipitation on Σ_16_PAHs is analyzed in more detail in the following section.

As can be seen in Table S2, only weak and statistically insignificant correlations (*p* < 0.05) were found between individual PAHs and wind field. In the case of gaseous pollutants, the highest R*s* of − 0.66 was observed between NO_×_ concentrations and wind direction, followed by NO_2_/V_d_ (− 0.59), PM_10_/V_d_ (− 0.34), SO_2_/V_d_ (− 0.31), and CO/V_d_ (− 0.29). This finding suggests that wind direction influences the variations in NO_×_, NO_2_ and SO_2_ concentrations. For wind speed, this factor was less correlated than wind direction related to almost all variables, excluding NO_2_ and NO_×_. The most plausible explanation for this is the impact of combined atmospheric processes i.e. large-scale transport, dispersion of particles from sources, and differences in atmospheric flow patterns. This is well in line with previous studies from China^15^ and Europe^1,10^, indicating the importance of considering a wider meteorological range (local and non-local variables), while analyzing variability of PAHs in the gas and particle phases.

In the present study, individual PAHs were positively correlated with SO_2_ and CO (*p* < 0.05, Table S2). In particular, SO_2_/PAH ratio exhibited *Rs* values greater than 0.60 for Phe, Flt, Pyr, and BaA, while CO/PAH ratio showed R*s* in the range of 0.47–0.66 for all PAHs except Naph, Ace and Acy. This finding suggests the same anthropogenic sources (i.e. fossil fuel combustion) and atmospheric processes. In contrast, almost all PAH isomers (excluding Acy) were anti-correlated with O_3_ concentrations, suggesting their chemical decomposition by heterogeneous reaction with ozone. Specifically, statistically significant and moderate correlations were found between ozone and Flu, Phe, BaP and Pyr. These results suggest photodegradation processes of PAHs in the particulate phase by ozone that could significantly affect PAH transformations, especially during summer and spring, when concentrations of O_3_ are high (mean = 76.3 and 66.8 µg m^−3^, respectively)^29^. Similar photochemical effects of ozone on particulate PAHs during the warm study period have been demonstrated in previous studies^13,15,16,49^.

Based on Table S2 it can be seen that there are correlations between 4-, 5- and 6-ring PAHs, with statistically significant *Rs* in the range of 0.75–0.99. The correlations between 2- and 3-ring PAHs (i.e. Ace and Acy) in the gas phase were significant (*p* < 0.05) and ranged from 0.65 to 0.82. Such a robust relationship can be explained by similar point, the area and mobile sources (i.e. residential/industrial combustion, traffic emissions, shipping activities) that are temporally variable. As mentioned above, physico-chemical processes in the atmosphere (i.e. photolysis, photochemical reactions, degradation by ozone, gas-to-particle partitioning, and atmospheric transport patterns) may also play a significant role in PAH variability, particularly during high pollution periods.

### The effect of precipitation and air mass transport on PAH levels

Precipitation is an important atmospheric process that drives the intra- and inter-annual trends of air pollutants. Recent work by Isokääntä et al*.*^50^ showed that wet deposition, including wet scavenging (below and in-cloud) and aqueous phase oxidation in-cloud, may strongly influence aerosol particle concentrations, size distribution and chemical composition. Thus, PAH chemistry and transformation are closely related to atmospheric conditions, including precipitation intensity, amount and frequency. In the present study, 14 daily precipitation episodes were selected to analyze their direct effects on the Σ_16_PAHs abundance. More specifically, to quantify the effect of precipitation on PAH levels, the mean concentrations of all compounds measured in daily samples during rain episodes were compared with their corresponding results before and after the precipitation event (Table S3). Additionally, Table S3 shows the Σ_16_PAH concentrations separately for the warm and cold seasons.

The results summarized in Table S3 show that the influence of precipitation on pollutants concentration led to ΣPAH losses from 21 to 83% depending on the meteorological situation (i.e. sampling period, precipitation amount, prevailing wind direction, air mass transport).

Among the daily episodes considered in this study, a maximum decrease of 83% in Σ_16_PAH concentrations was observed during the warm period. This particular case was registered for days with air pollutants transport over the European countries (e.g. the UK, northern France, Germany, Poland) and a relatively low precipitation amount (2.4 mm), indicating that meteorological conditions affected PAH wet scavenging in the target Baltic area (Table S3, no.5). By contrast, high precipitation amounts on 11 Jun 2019 (29.9 mm) reduced Σ_16_PAH by 51%. On that day, the FLEXTRA backward trajectory model revealed that air masses primarily came from S and SE sectors (Table S3, no.1). A decrease in Σ_16_PAH concentrations identified on 7 May 2020, 5 Oct 2019 and 9 Jul 2019 was also attributed to high precipitation amount (22.3 mm, 19.0 mm, and 21.0 mm), meanwhile, values of PAH losses were slightly lower compared to case study no.1 and accounted for 44%, 42%, and 21%, respectively (Table S3, no.4,6,14). It should be highlighted that these cases were mainly attributed to northern flow from Scandinavia (marine sector), indicating that less polluted air masses were transported to the receptor domain.

During the cold season, the highest Σ_16_PAH loss (79%) by wet deposition was found on 11 January 2020, while slightly lower declines were observed on 10 and 12 February 2020 (71%). This was likely due to high ΣPAH concentrations in the ambient air a day before and/or during the rainfalls (range from 21.3 to 73.8 ng m^-3^), and relatively low precipitation rate (snow) that did not exceed 3.0, 6.8, and 8.4 mm, respectively (Table S3, no.10,12,13). Again, from these results, one could conclude that precipitation episodes contribute to the reduction of PAH levels reported in this study. As shown in Table S3, the predominant westerly air mass transport was identified for these days, indicating a large impact of local/regional combustion-related sources (i.e. coal-fired power plants, commercial and domestic boilers) on PAH levels during the cold season.

Drotikova et al*.*^12,51^ showed that Flt and Pyr emitted in the coastal region of Norway partly originated from shipping activities. A mass ratio of *Flt/(Flt* + *Pyr)* in the range of 0.31–0.42 can be used as a marker of primary shipping emissions^52^. As shown in Fig. S3, the mean *Flt/(Flt* + *Pyr)* in our coastal urban region was higher than 0.50 (range from 0.23 to 0.76), which implies that residential/industrial emissions belong to major primary PAH sources. However, almost 20% of all collected samples demonstrated the contribution of marine fuel combustion and petrol emissions to PAH sources. This finding suggests that shipping activities have an impact on PAH burden, even though other anthropogenic inputs such as fuel combustion for heating and vehicle emissions play a predominant role in this region.

Preliminary results from BaP-oriented studies by Staniszewska et al*.*^53^ showed that daily heavy precipitation events (> 28.6 mm) may lead to a decrease in 5-ring PAH concentrations in aerosol samples collected during or a day after the rainfalls. Our study demonstrates that also daily light/medium precipitation events can impact PAH burden. As shown in Table S2, precipitation was negatively correlated with Ace, Flu, Flt, Chry, BbF, BkF, BaP, DahA, BghiP, and IcdP, while positive correlations were found for 2-ring and 3-ring (only Acy) PAHs. Therefore, there is a strong need to fully characterize the effect of precipitation on inter-annual differences in PAH levels and composition, and their transfer from the atmosphere to the water/soil system.

### Potential health risk assessment of PAHs

The USEPA criteria for ILCR can provide a simple and relevant tool for comprehensive health risk assessment^7,32^. Generally, ILCR values (1) below 10^−6^, (2) in the range 10^−6^–10^−4^, and (3) higher than 10^−4^ represent low/acceptable, moderate/potential, and high/serious public health problems, respectively. The results of this study indicate the potential public health risks due to daily PAH inhalation exposure. However, it is noteworthy to mention that as shown in Figs. 3 and 4, most of the carcinogenic compounds used for calculation of ILCR values were detected in the particle phase. Table 1 compares the statistical results of ILCR based on three different population groups concerning the cold and the warm season. The ratios of 95–5% percentile values for total ILCR in adult males, adult females and children were not significantly different (1.02/0.02, 1.33/0.01, 1.42/0.01, respectively).

**Table 1:** Statistical summary of ILCR (× 10^−6^) calculated for different population groups during the cold season, the warm season and the entire study period (Total).

Group	Period	Mean ± SD	Min	Max	5% percentile	95% percentile
Adult males (× 10^−6^)	Warm	0.11 ± 0.17	0.01*	0.91	0.01	0.53
	Cold	0.43 ± 0.42	0.01	1.43	0.02	1.25
	Total	0.23 ± 0.33	0.01*	1.43	0.01	1.02
Adult females (× 10^−6^)	Warm	0.14 ± 0.22	0.02*	1.18	0.01	0.69
	Cold	0.57 ± 0.55	0.02	1.87	0.02	1.64
	Total	0.30 ± 0.43	0.02*	1.87	0.01	1.33
Children (× 10^−6^)	Warm	0.15 ± 0.24	0.02*	1.27	0.01	0.74
	Cold	0.61 ± 0.58	0.02	2.00	0.02	1.75
	Total	0.32 ± 0.46	0.02*	2.00	0.01	1.42

Data marked as (*) show values × 10^−8^.

The total mean ILCR ranged from 0.23 ± 0.33 × 10^−6^ in the adult males group to 0.32 ± 0.46 × 10^−6^ in children. It can be seen in Table 1 that the mean ILCR was higher in adult females than that in adult males during the cold (0.57 × 10^−6^ vs. 0.43 × 10^−6^) and the warm season (0.14 × 10^−6^ vs. 0.11 × 10^−6^). Moreover, ILCR calculated for children was substantially higher during the cold season (0.61 ± 0.58 × 10^−6^) relative to the warm season (0.15 ± 0.24 × 10^−6^). More recent studies^31,32^ found that total inhalation intakes of PAHs can be more than 2 orders of magnitude higher during the cold season due to elevated concentrations of BaP and DahA. It should be highlighted that a similar scheme was observed in this study, indicating relatively high emission of carcinogenic PAHs during winter. Moreover, other carcinogenic PAHs (i.e. BaA, BkF, BbF, BghiP, Chry) also revealed a substantially higher contribution to the total PAHs during the cold season than those measured during the warm season (Fig. 4).

**Figure 5:**
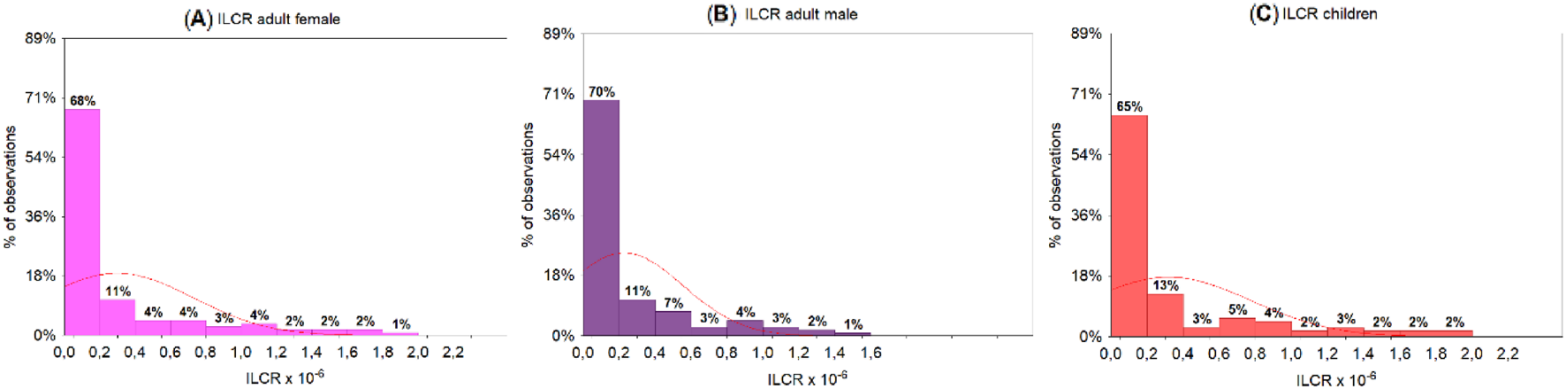
Histogram plots of ILCR frequency distribution (× 10^−6^) in three different population groups: (**A**) adult females, (**B**) adult males, and (**C**) children.

As mentioned above, winter coal combustion in domestic units and the use of fossil fuels by commercial sectors are significant sources of PAHs in Poland. Additionally, the presence of specific conditions, which favours the accumulation of pollutants during winter pollution episodes (i.e. low wind speed, low temperature, thermal inversion, foggy situations, low mixing layer height, and low solar radiation) may cause preferential pathways for gas to PM conversion and consequently for other chemical PAH transformation that enhance high levels of carcinogenic compounds in the atmosphere. Figure 5 shows histogram plots of ILCR values calculated using Eq. (3). It can be seen that ILCRs distribution had a remarkedly similar log-normal shape, with clear frequency tails towards the highest values related to all groups. The highest relative frequency of 70%, 68%, and 65% was found for ILCR < 0.2 × 10^−6^ respectively in adult males, adult females, and children. The ILCR values in Gdynia were in a similar range to those measured in Monte Velho, Portugal (4.4 × 10^−6^)^10^, and Eskişehair, Turkey (1.02–1.74 × 10^−6^)^32^, but much lower than those determined in Wangdu, China (4.39 × 10^−3^)^35^. Previous PAH measurements in Singapore^7^ reported ILCR values lower than the minimum acceptable level, with an increasing trend of potential high cancer risk to adults and children during haze conditions.

The BaP equivalent (BaP_eq_) toxicity in this study varied between 0.01 to 6.05 ng m^−3^, with a mean value of 0.96 ng m^−3^. Additionally, the BaP_eq_ concentrations showed lower values during the warm season (mean = 0.45 ± 0.72 ng m^−3^) than during the cold season (mean = 1.84 ± 1.77 ng m^−3^), suggesting the significance of local/regional emissions. Our previous study^25,28,29^ have observed similar processes for toxic trace elements and organics in the fine fraction of particulate matter. Similar results were derived for carcinogenic hydrocarbons in other worldwide locations^10,31,32^. It is therefore concluded that PAH emissions in this region (southern Baltic) should be at least limited during the winter season to mitigate poor air quality.

## Conclusions and atmospheric implications

The simultaneous measurements of Σ_16_PAHs in the gas and particle phases, the first of this kind in this region, report on a wide range of possible interactions from different environmental factors which have to be considered when evaluating the levels, and seasonal trends of aromatic hydrocarbons.

The current study finds that local and regional anthropogenic sources significantly affect PAH concentrations in the gas and particle phases, their distribution and seasonal profiles. A contribution of 5- and 6-ring isomers to the total PAHs increased significantly in winter, which was respectively almost 10 and 5 times higher than results registered in summer.

This study highlights the impact of meteorology on PAHs in gas and particle phases. Most of the analyzed cases showed that concentrations of PAH congeners declined after rain episodes, particularly during the cold season. This work shows that precipitation substantially affects PAH transformations in the ambient air. Precipitation-driven loss of ΣPAH was very variable depending on the study period, PAH levels, precipitation characteristics and transport pathways.

For different seasons, higher values of BaP_eq_ concentrations were observed in autumn and winter (mean = 1.84 ± 1.77 ng m^−3^), suggesting relatively higher local/regional emissions of toxic PAHs in the cold season compared to the warm one. The estimated ILCRs were higher in children than the corresponding values observed in both adult groups, highlighting the need for better pollution-control policies in this region.

## Supplementary Information

Supplementary Information.

## Data Availability

All data generated and analyzed during this study are included in this article (and its supplementary files).
