# Supplementary material for: Compositional and seasonal differences of gas and particle phase polycyclic aromatic hydrocarbons (PAHs) over the southern Baltic Sea coast
Source: Sci Rep. 2022 Dec 5;12:21005. doi: 10.1038/s41598-022-25666-5 (PMC9723112; doi:10.1038/s41598-022-25666-5)
Supplement: Supplementary file 1 — Supplementary Information. [file 41598_2022_25666_MOESM1_ESM.pdf]

Supplement of

Compositional and seasonal differences of gas and particle phase polycyclic aromatic hydrocarbons (PAHs) over the southern Baltic Sea coast

Patrycja Siudek

Correspondence to: Patrycja Siudek (patrycja.siudek@imgw.pl)

Figure S1 The sampling site (**SP**) in Gdynia, Poland. Dots represent major local/regional PAH sources: coal-fired power plant (red), port and docks area (blue), petrochemical refinery and plants (green), and municipal solid waste recycling units (yellow). The ESRI ArcGIS Desktop software v. 9.3 was used to process the data and create a map (on left). The illustration (on right) was prepared using a map data ©2022 Google.

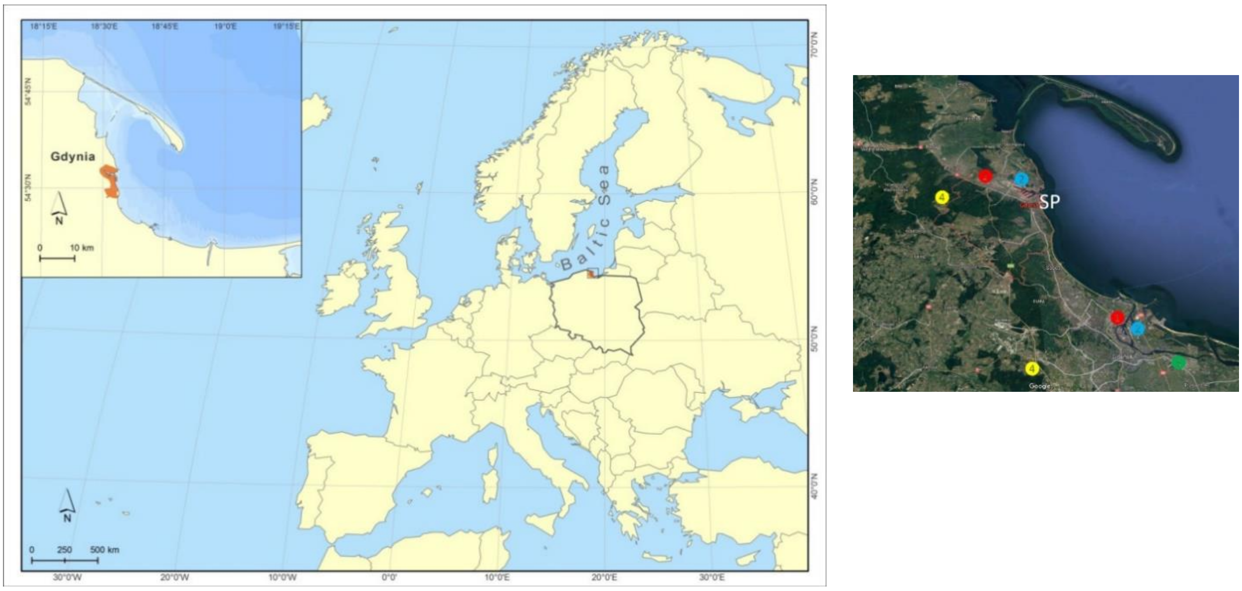

Figure S2 Daily variation of Σ_{16}PAHs concentration (ng m^{-3}) in gas and particle phase for each month of the 2019-2020 sampling period

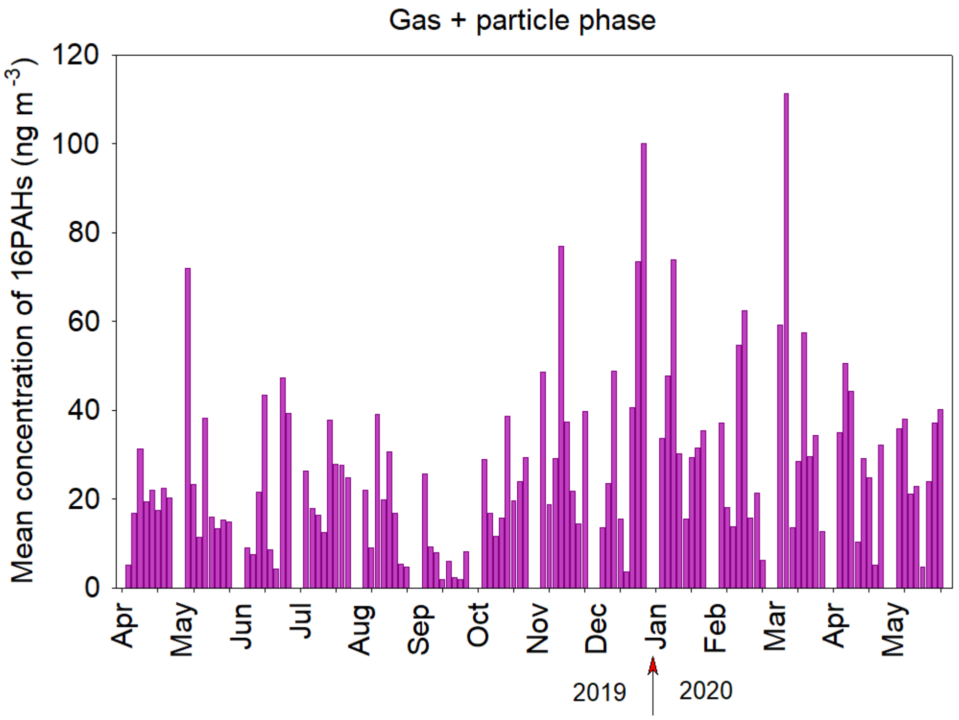

Figure 3S Seasonal variation of diagnostic ratios of different PAHs. The error bars indicate one standard deviation.

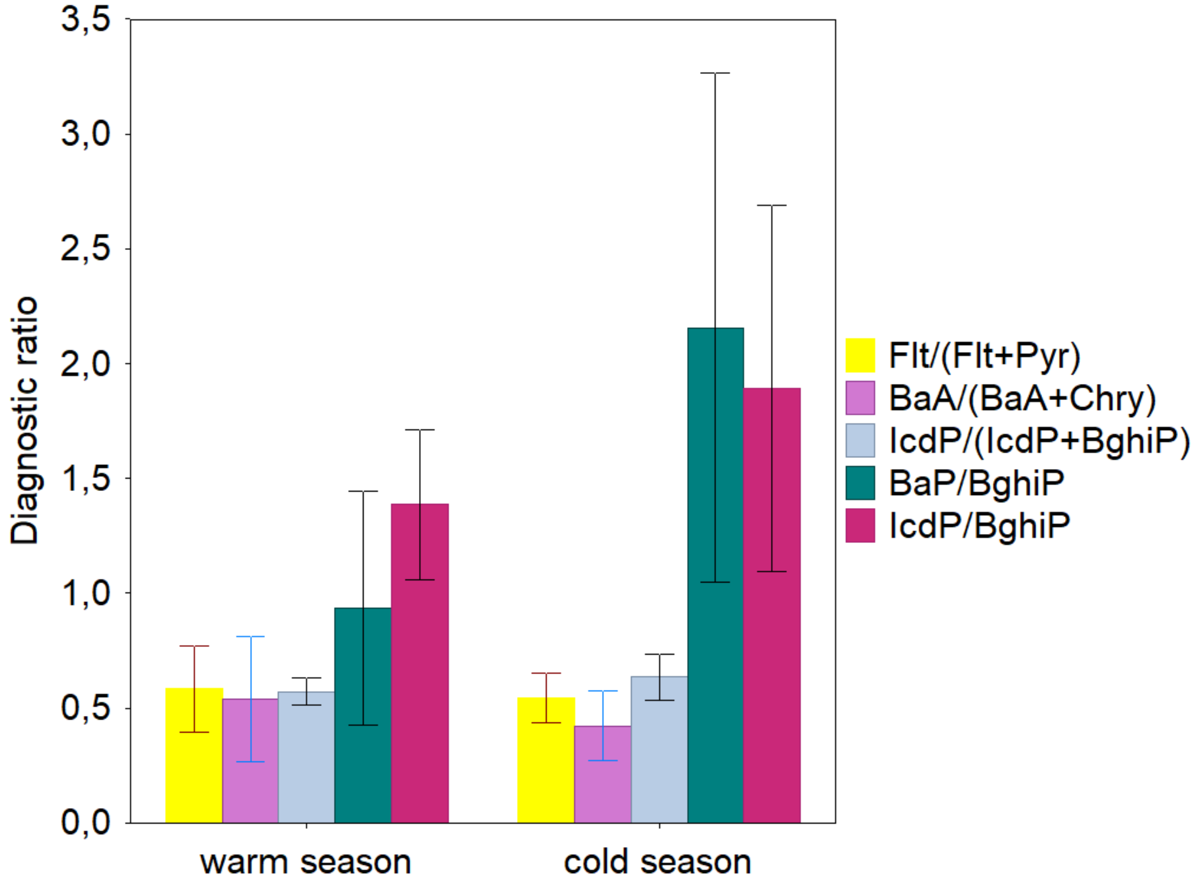

Table 1S Characteristics of PAH isomers. TEF_i – toxic equivalent factor

	Ring	TEF_i
Nap	2	0.001
Ace	3	0.001
Acy	3	0.001
Flu	3	0.001
Phe	3	0.001
Ant	3	0.01
Flt	4	0.001
Pyr	4	0.001
BaA	4	0.1
Chry	4	0.01
BaP	5	1
BbF	5	0.1
BkF	5	0.1
DahA	5	1
BhgiP	6	0.01
IcdP	6	0.1

Table S2 Spearman's correlation matrix (Rs value) between concentrations of PAH components, gaseous species and meteorological parameters during the field campaigns. The concentrations of PAHs in gaseous and particulate phases are analyzed separately.

The abbreviations are as follows: O_3 – ozone concentration, NO_x - nitrogen oxides concentration, NO_2 – nitrogen dioxide concentration, SO_2 – sulfur dioxide concentration, CO – carbon monoxide, PM_{10} – coarse mode particles, T – ambient air temperature in °C, Rh – relative humidity in %, V_s – wind speed in m/s, V_d – prevailing wind direction in degree, Pa – precipitation in mm

	Pa	T	Rh	V_d	V_s	SO_2	NO_2	O_3	CO	NO_x	PM_{10}	Nap	Ace	Acy	Flu	Phe	Ant	Flt	Pyr	BaA	Chry	BbF	BkF	BaP	DahA	BghiP	IcdP
**Pa**	1.00																										
**T**	0.04	1.00																									
**Rh**	0.39	-0.15	1.00																								
**V_d**	0.42	-0.01	0.08	1.00																							
**V_s**	0.11	-0.11	0.05	0.24	1.00																						
**SO_2**	-0.37	-0.63	-0.06	-0.31	-0.13	1.00																					
**NO_2**	-0.37	-0.15	0.23	-0.59	-0.30	0.43	1.00																				
**O_3**	-0.10	0.30	-0.58	0.02	0.00	-0.24	-0.48	1.00																			
**CO**	-0.19	-0.64	0.02	-0.29	-0.14	0.71	0.47	-0.44	1.00																		
**NO_x**	-0.38	-0.24	0.26	-0.66	-0.27	0.46	0.98	-0.49	0.53	1.00																	
**PM_{10}**	-0.23	0.17	-0.05	-0.34	-0.15	0.06	0.29	0.10	0.10	0,39	1.00																
**Nap**	0.03	-0.29	0.09	0.12	0.07	0.22	-0.02	-0.12	0.22	0.00	-0.01	1.00															
**Ace**	-0.01	-0.20	0.13	0.12	-0.05	0.12	-0.04	-0.15	0.20	-0.01	0.03	0.65	1.00														
**Acy**	0.12	-0.11	-0.04	0.09	0.04	0.06	-0.12	0.09	0.01	-0.01	0.15	0.77	0.54	1.00													
**Flu**	-0.08	-0.48	0.21	0.22	0.00	0.57	0.25	-0.50	0.59	0.28	-0.03	0.82	0.57	0.88	1.00												
**Phe**	-0.29	-0.60	0.29	0.24	0.03	0.64	0.41	-0.49	0.64	0.44	0.11	0.75	0.48	0.82	0.77	1.00											
**Ant**	-0.30	-0.51	0.11	0.22	0.04	0.56	0.39	-0.41	0.53	0.45	0.16	0.40	0.42	0.38	0.67	0.82	1.00										
**Flt**	-0.23	-0.60	0.14	-0.16	0.02	0.60	0.32	-0.33	0.57	0.34	0.19	0.47	0.38	0.49	0.65	0.82	0.77	1.00									
**Pyr**	-0.28	-0.71	0.27	0.07	0.02	0.71	0.45	-0.46	0.66	0.54	0.14	0.55	0.45	0.46	0.71	0.92	0.82	0.82	1.00								
**BaA**	-0.17	-0.57	0.16	0.10	-0.04	0.62	0.33	-0.38	0.50	0.30	0.02	0.29	0.28	0.19	0.64	0.78	0.86	0.75	0.79	1.00							
**Chry**	-0.15	-0.70	0.28	-0.02	-0.01	0.57	0.38	-0.34	0.53	0.51	0.25	0.36	0.39	0.20	0.65	0.85	0.82	0.82	0.93	0.85	1.00						
**BbF**	-0.10	-0.62	0.36	-0.06	-0.06	0.46	0.43	-0.43	0.50	0.63	0.29	0.27	0.36	0.15	0.61	0.82	0.78	0.77	0.87	0.80	0.93	1.00					
**BkF**	-0.18	-0.66	0.30	-0.06	-0.07	0.54	0.40	-0.35	0.50	0.54	0.30	0.23	0.34	0.10	0.64	0.86	0.83	0.84	0.92	0.85	0.99	0.95	1.00				
**BaP**	-0.16	-0.65	0.31	-0.02	-0.05	0.53	0.40	-0.48	0.56	0.52	0.20	0.23	0.33	0.06	0.68	0.88	0.83	0.82	0.91	0.84	0.96	0.94	0.96	1.00			
**DahA**	-0.14	-0.61	0.23	-0.10	0.00	0.50	0.33	-0.42	0.51	0.38	0.18	0.31	0.31	0.11	0.63	0.82	0.84	0.84	0.82	0.85	0.87	0.84	0.88	0.89	1.00		
**BghiP**	-0.17	-0.65	0.24	-0.07	-0.04	0.54	0.36	-0.29	0.47	0.46	0.25	0.22	0.33	0.09	0.62	0.83	0.82	0.82	0.89	0.86	0.97	0.93	0.98	0.94	0.85	1.00	
**IcdP**	-0.16	-0.63	0.33	-0.07	-0.06	0.53	0.39	-0.34	0.50	0.54	0.31	0.30	0.36	0.14	0.65	0.85	0.81	0.82	0.91	0.84	0.97	0.94	0.98	0.96	0.88	0.96	1.00

Table S3 Summary of 14 case studies on the impact of daily precipitation on Σ_{16}PAH concentrations over the study region in Gdynia, including the backward trajectory analysis and percentage of PAH loss for each case. The cold season is shown as shaded plots, precipitation amount represents pink bars and concentrations are blue dots. The maps of air mass backward trajectories were prepared using the Lagrangian FLEXTRA trajectory model (projects.nilu.no/ccc/trajectories).

No.	Case study	Mean concentration of $\Sigma_{16}PAH_{g+p}$ (ng m^{-3}) and precipitation amount (mm) before, during, and after the precipitation episode	Backward trajectories<br>FLEXTRA 5.0 trajectory model using the European Centre for Medium-Range Forecasts (ECMWF)<br>operational data set ERA5 meteorological data at 0.25° horizontal resolution	Σ_{16}PAH<br>loss (%)
**1.**	**11 Jun 2019**	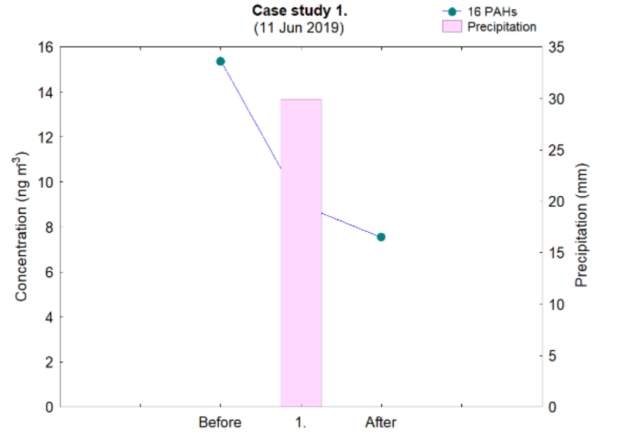	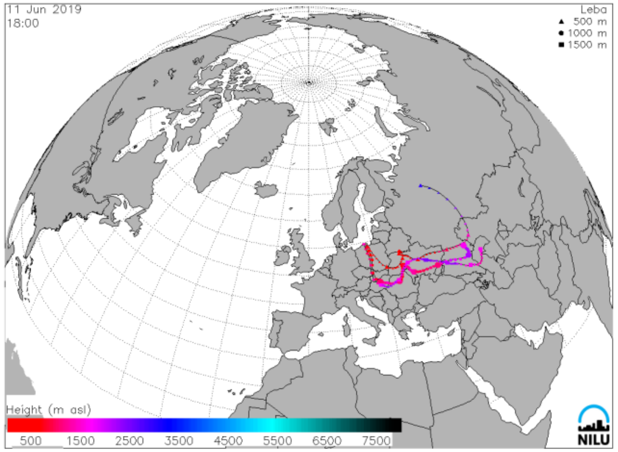	**51**

**2.**	**14 Jun 2019**	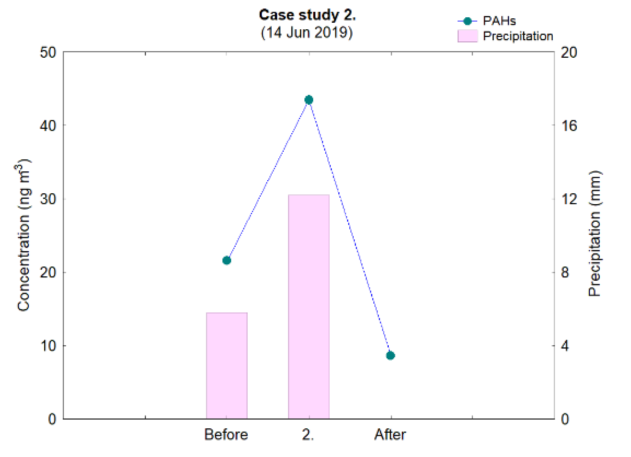	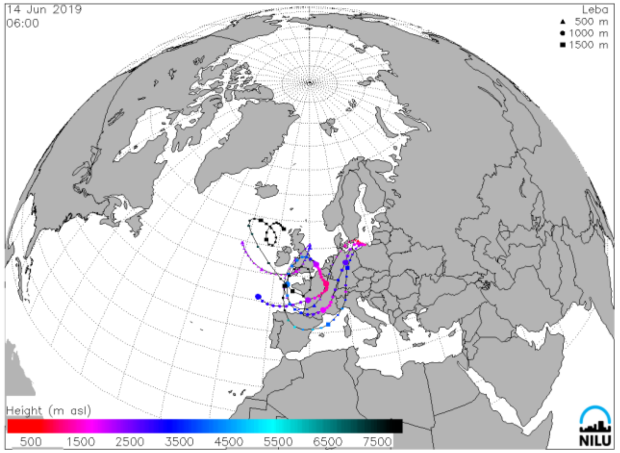	**60**
**3.**	**02 Jul 2019**	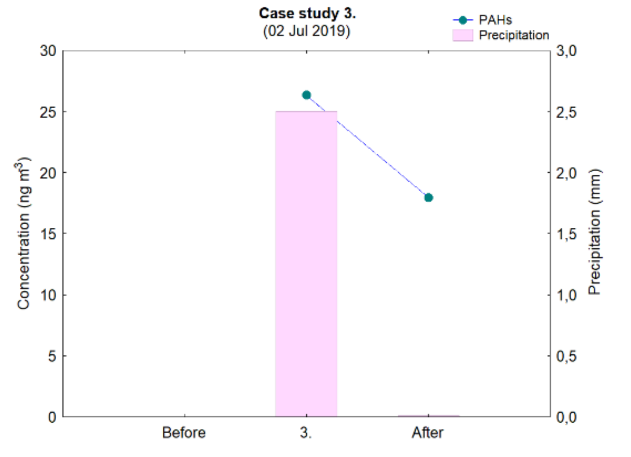	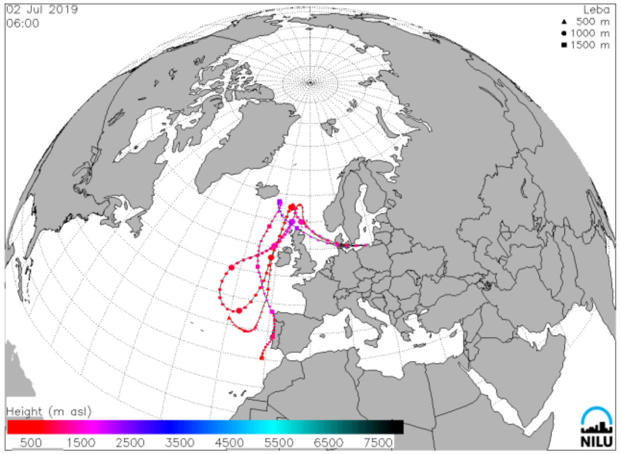	**32**

**4.**	**09 Jul 2019**	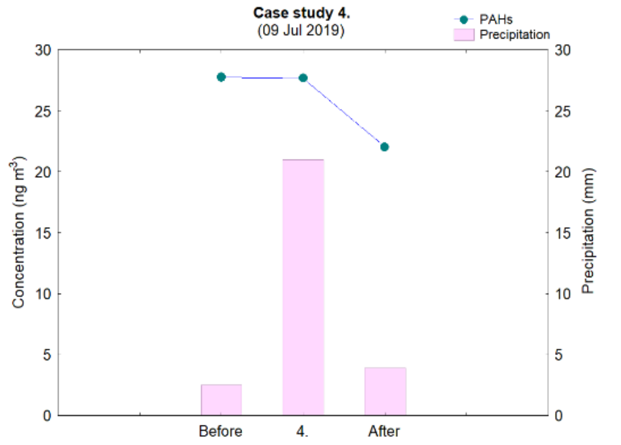	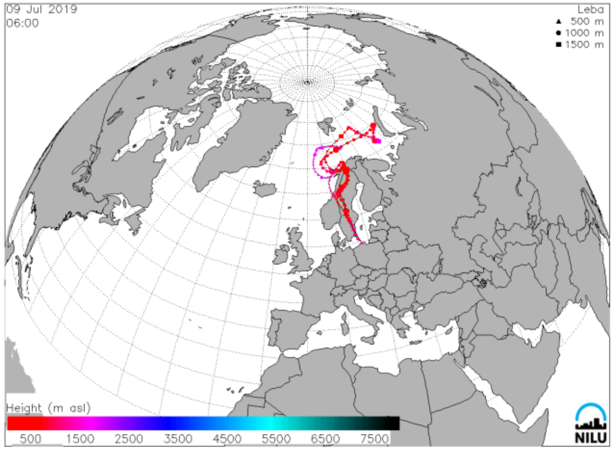	**21**
**5.**	**11 Aug 2019**	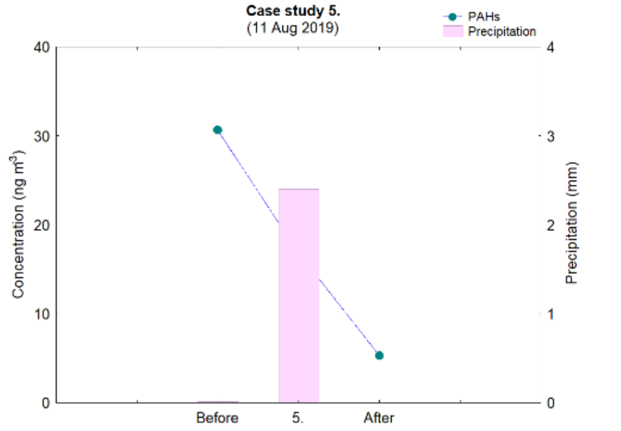	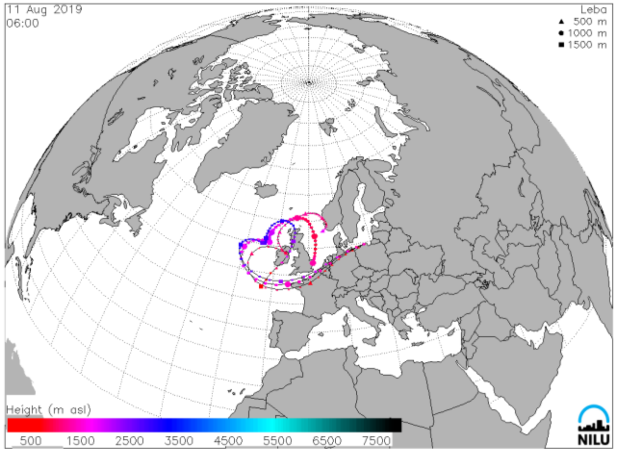	**83**

**6.**	**05 Oct 2019**	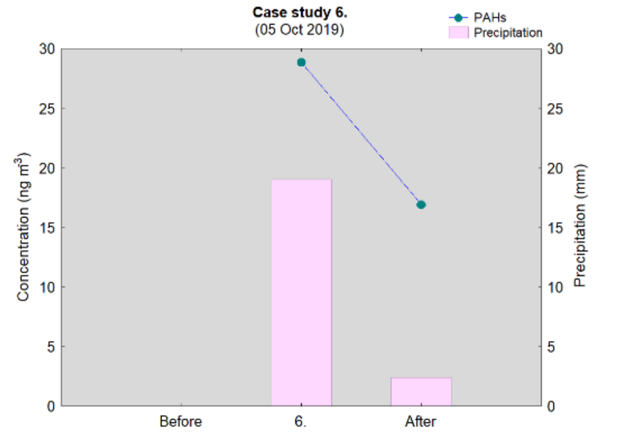	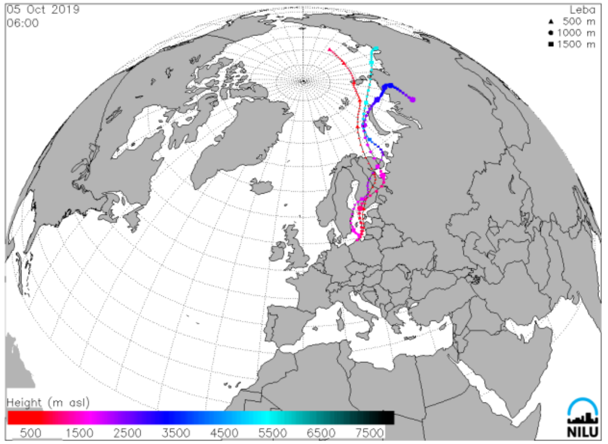	42
**7.**	**05 Nov 2019**	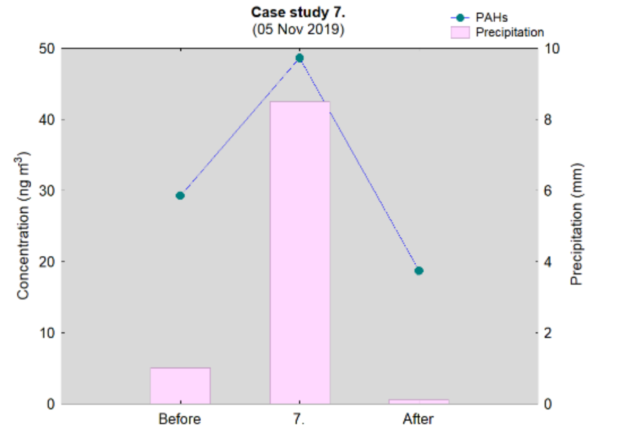	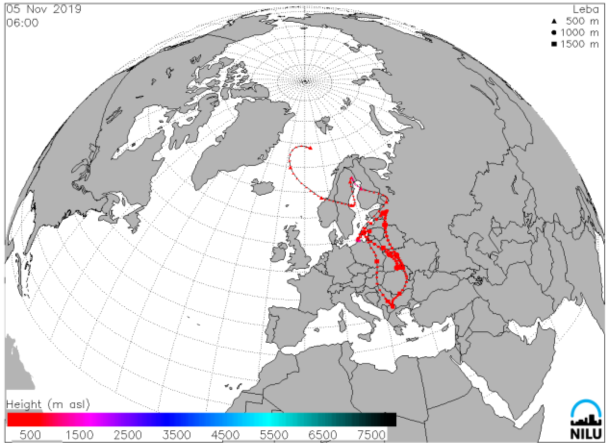	36

**8.**	**11 Nov 2019**	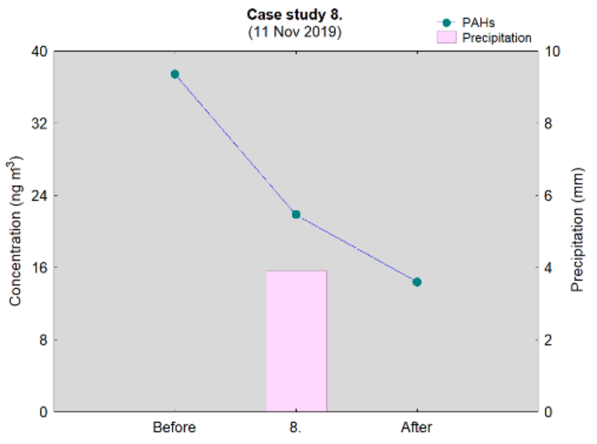	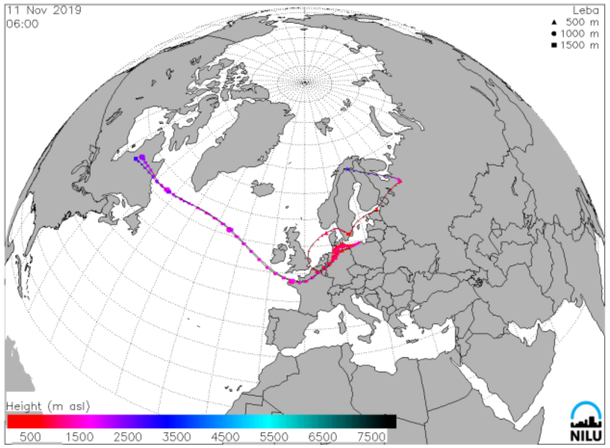	62
**9.**	**13 Nov 2019**	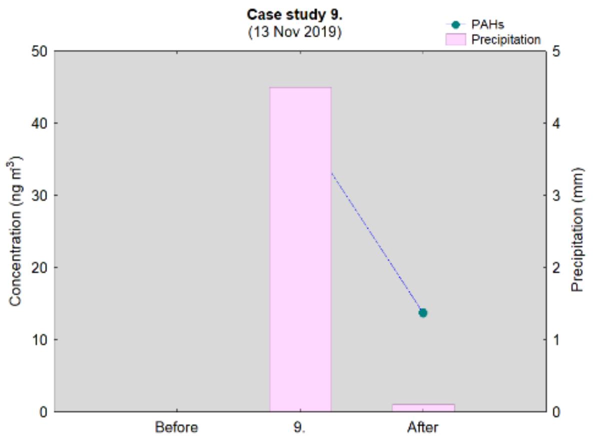	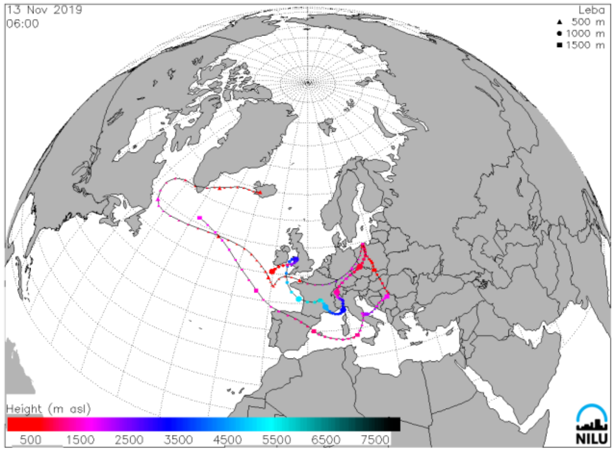	66

**10.**	**11 Jan 2020**	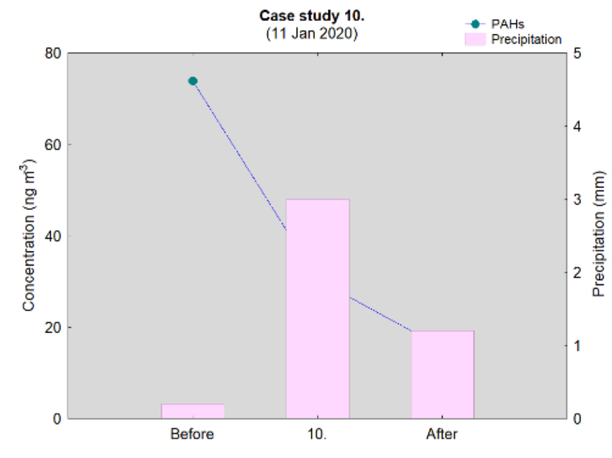	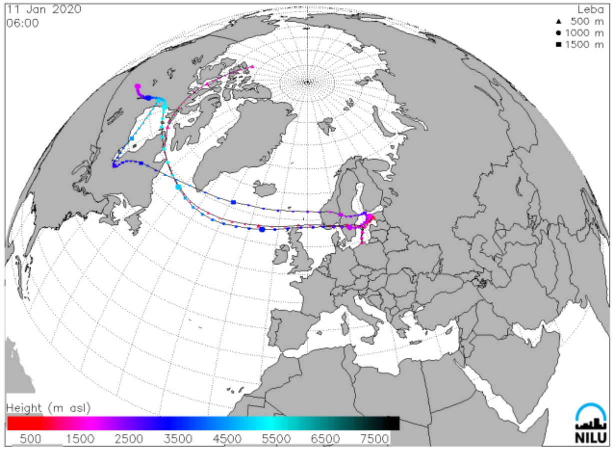	79
**11.**	**05 Feb 2020**	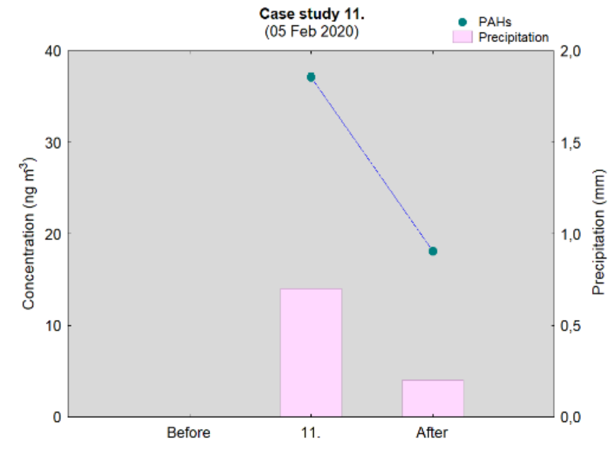	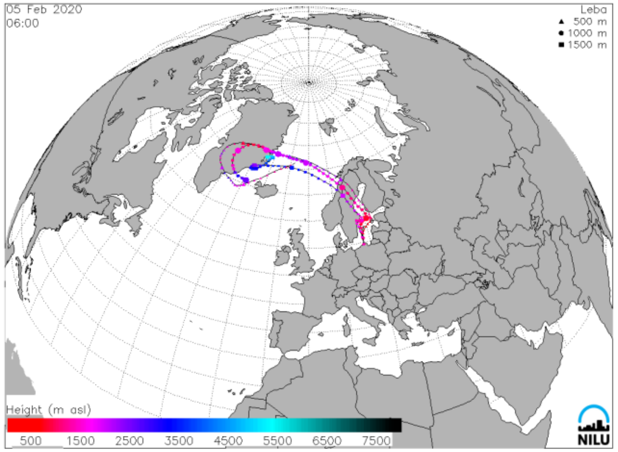	51

<table>
<tr>
<td>12.</td>
<td>10 Feb 2020</td>
<td>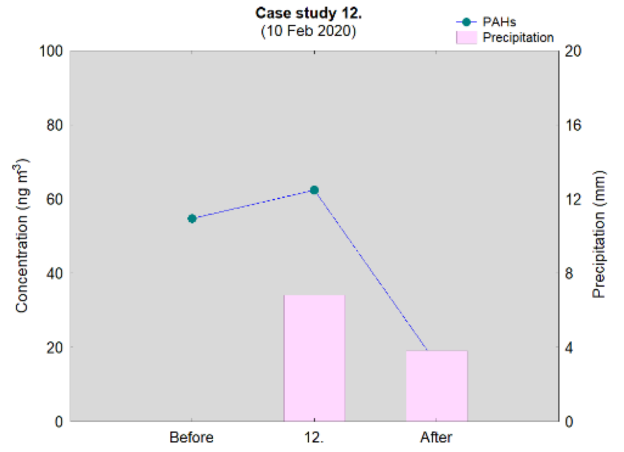
</td>
<td>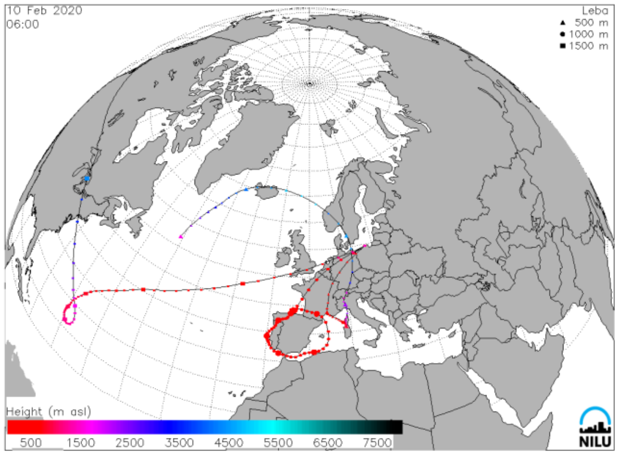
</td>
<td>71</td>
</tr>
<tr>
<td>13.</td>
<td>12 Feb 2020</td>
<td>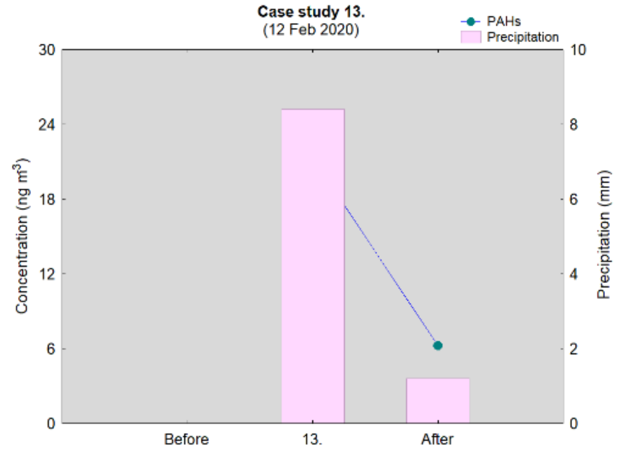
</td>
<td>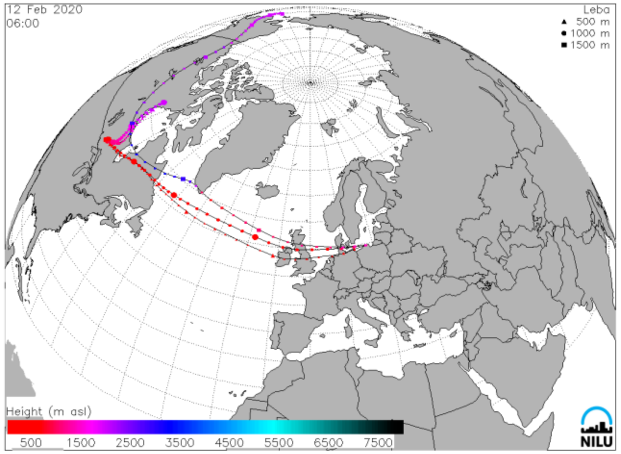
</td>
<td>71</td>
</tr>
</table>

**14.**	**07 May 2020**	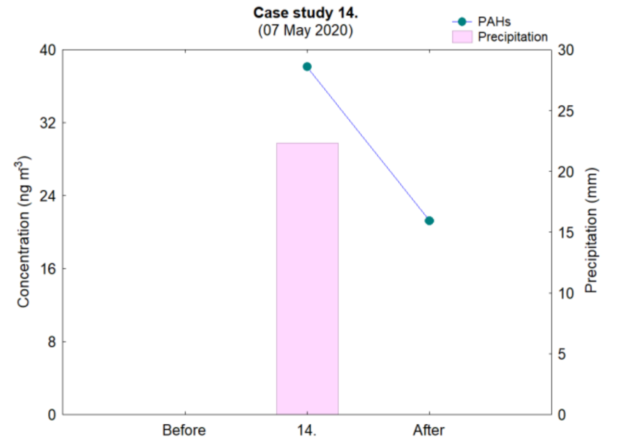	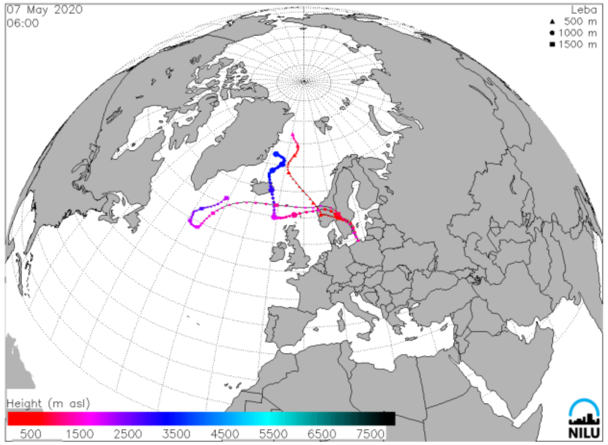	44
